# Supplementary material for: Evaluation and Validation of the Limited Sampling Strategy of Polymyxin B in Patients with Multidrug-Resistant Gram-Negative Infection
Source: Pharmaceutics. 2022 Oct 28;14(11):2323. doi: 10.3390/pharmaceutics14112323 (PMC9698835; doi:10.3390/pharmaceutics14112323)
Supplement: Supplementary file 1 [file pharmaceutics-14-02323-s001.zip › pharmaceutics-1918004-supplementary.pdf]

Supplementary Material

1 Supplementary Figures and Tables

1.1 Supplementary Figures

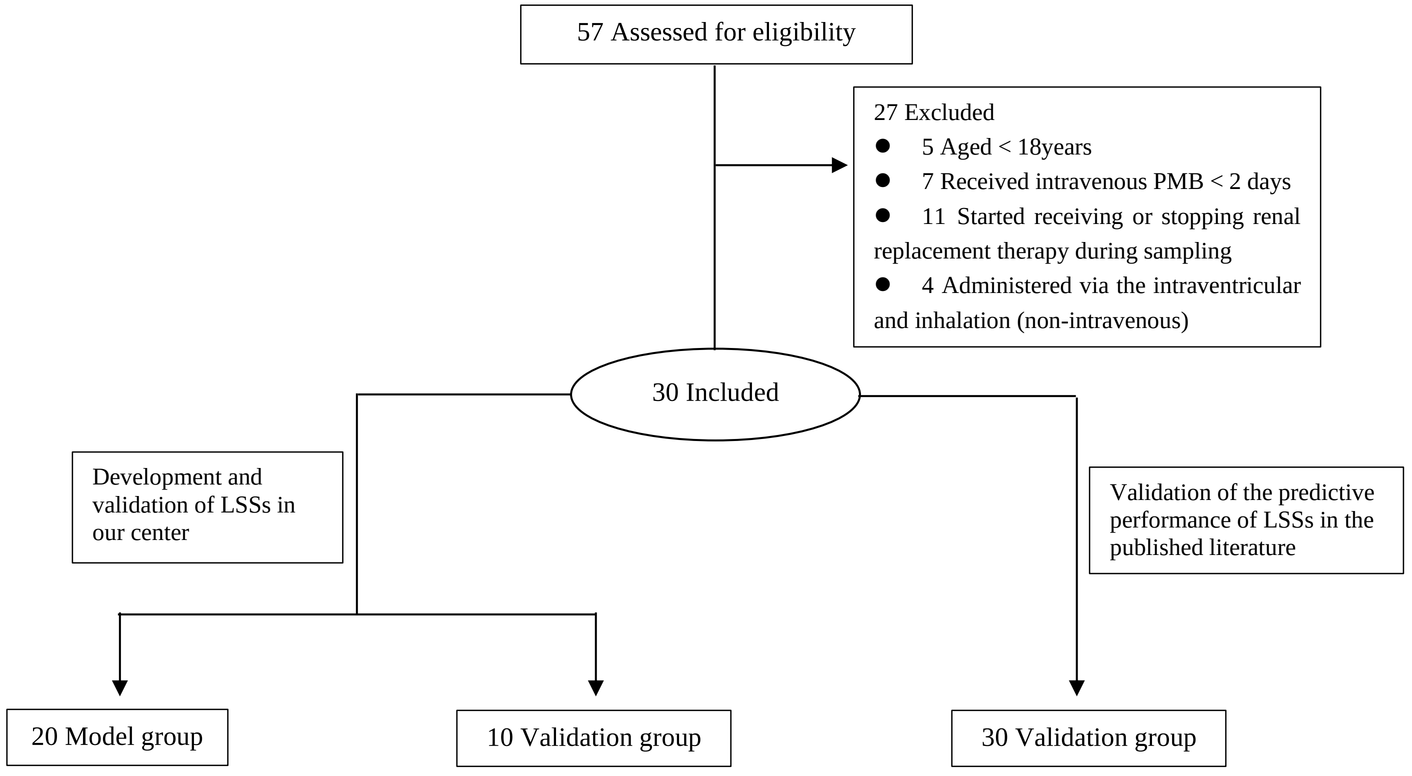

Figure S1. Study Flow Diagram.

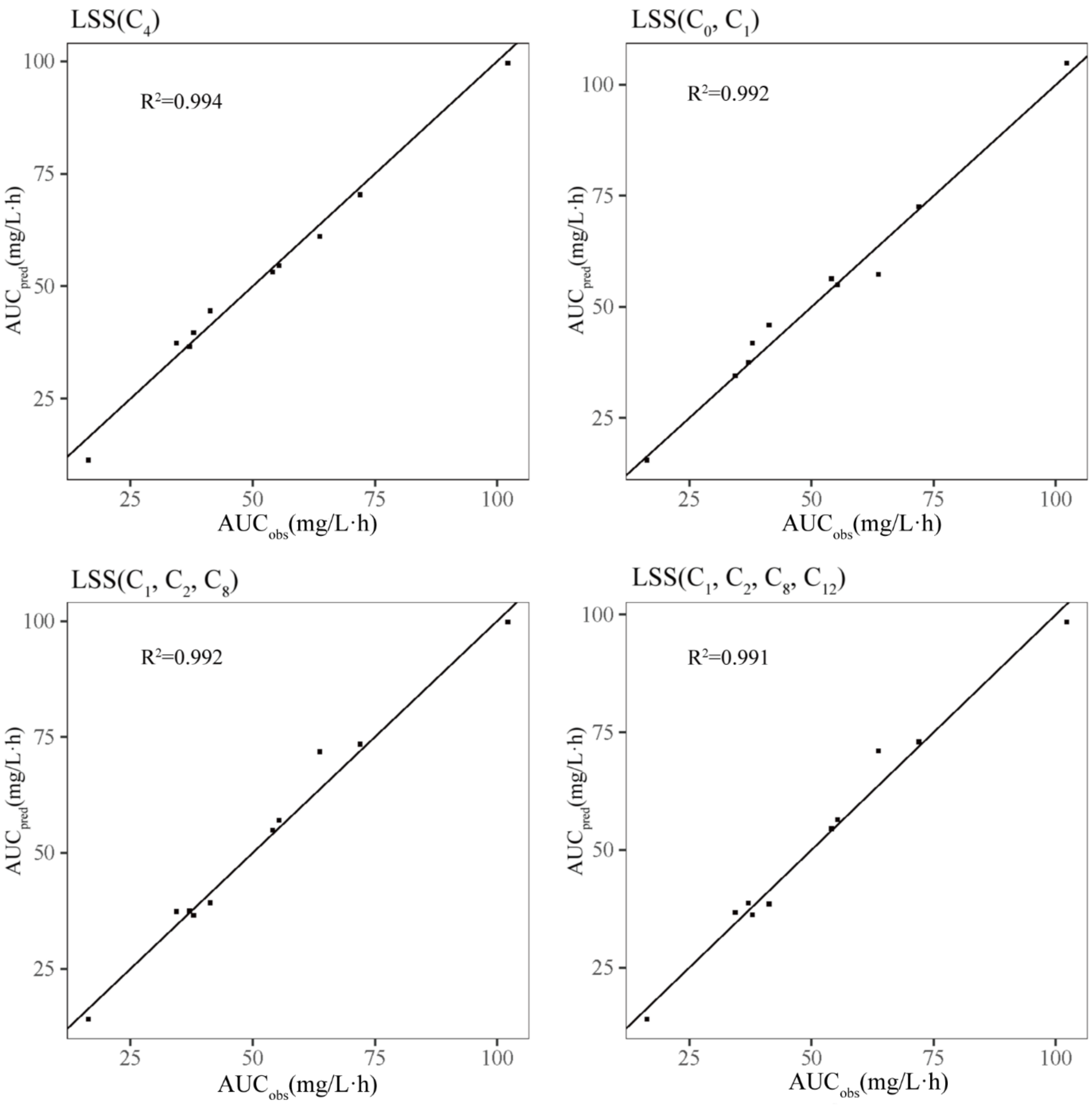

Figure S2. Correlation between observed area under the (0-24 h) concentration-time curve (AUC_{obs}) and predicted area under the (0-24 h) concentration-time curve (AUC_{pred}). AUC_{pred} were estimated from LSS C_4, (C_0, C_1), (C_1, C_2, C_8), and (C_1, C_2, C_4, C_8) in Our Center.

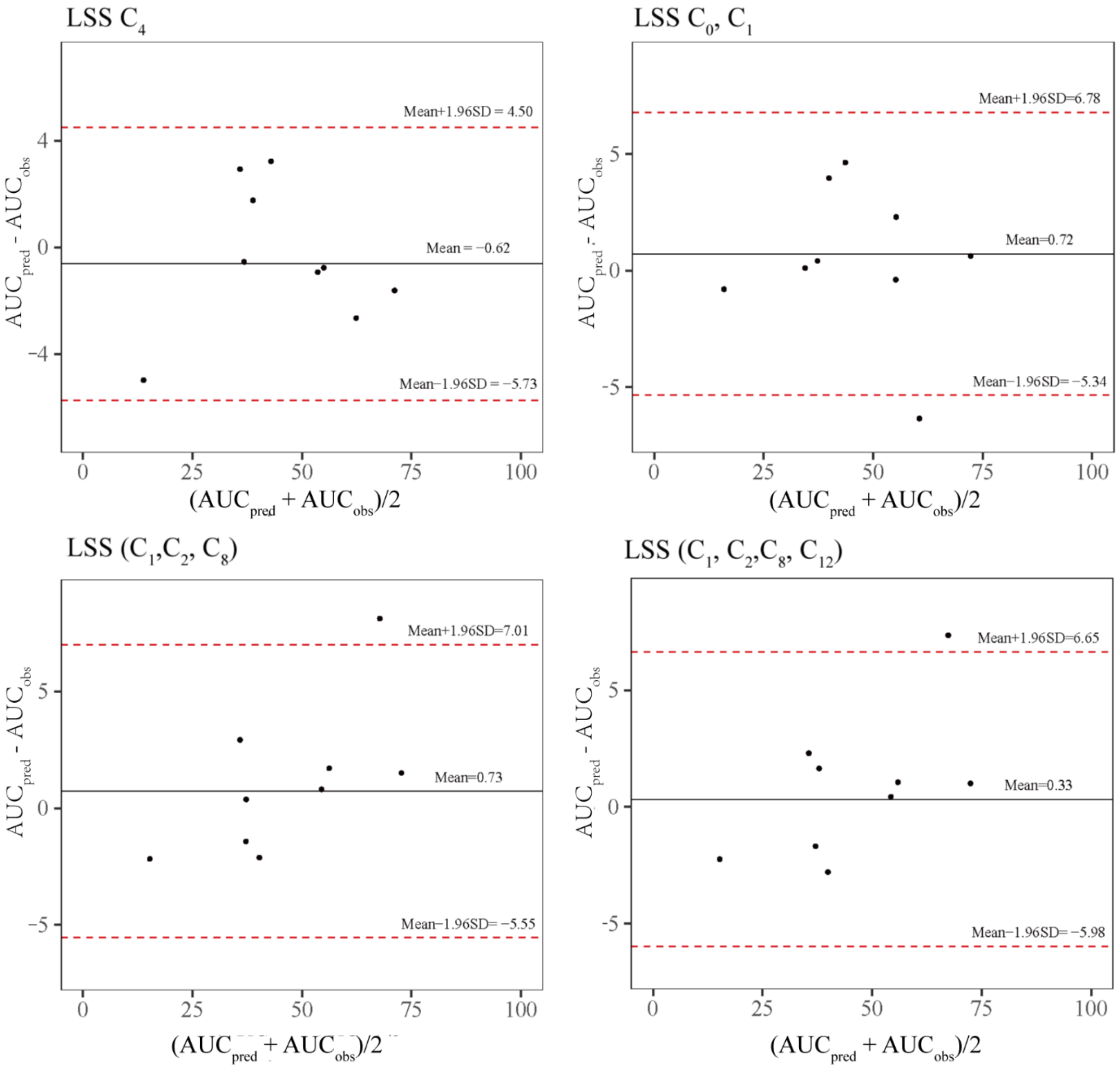

Figure S3. Bland-Altman plots of the relative difference between AUC_{pred} and AUC_{obs} versus the average of AUC_{pred} and AUC_{obs}. AUC_{pred} were estimated from LSS C_4, (C_0, C_1), (C_1, C_2, C_8), and (C_1, C_2, C_4, C_8) in our center.

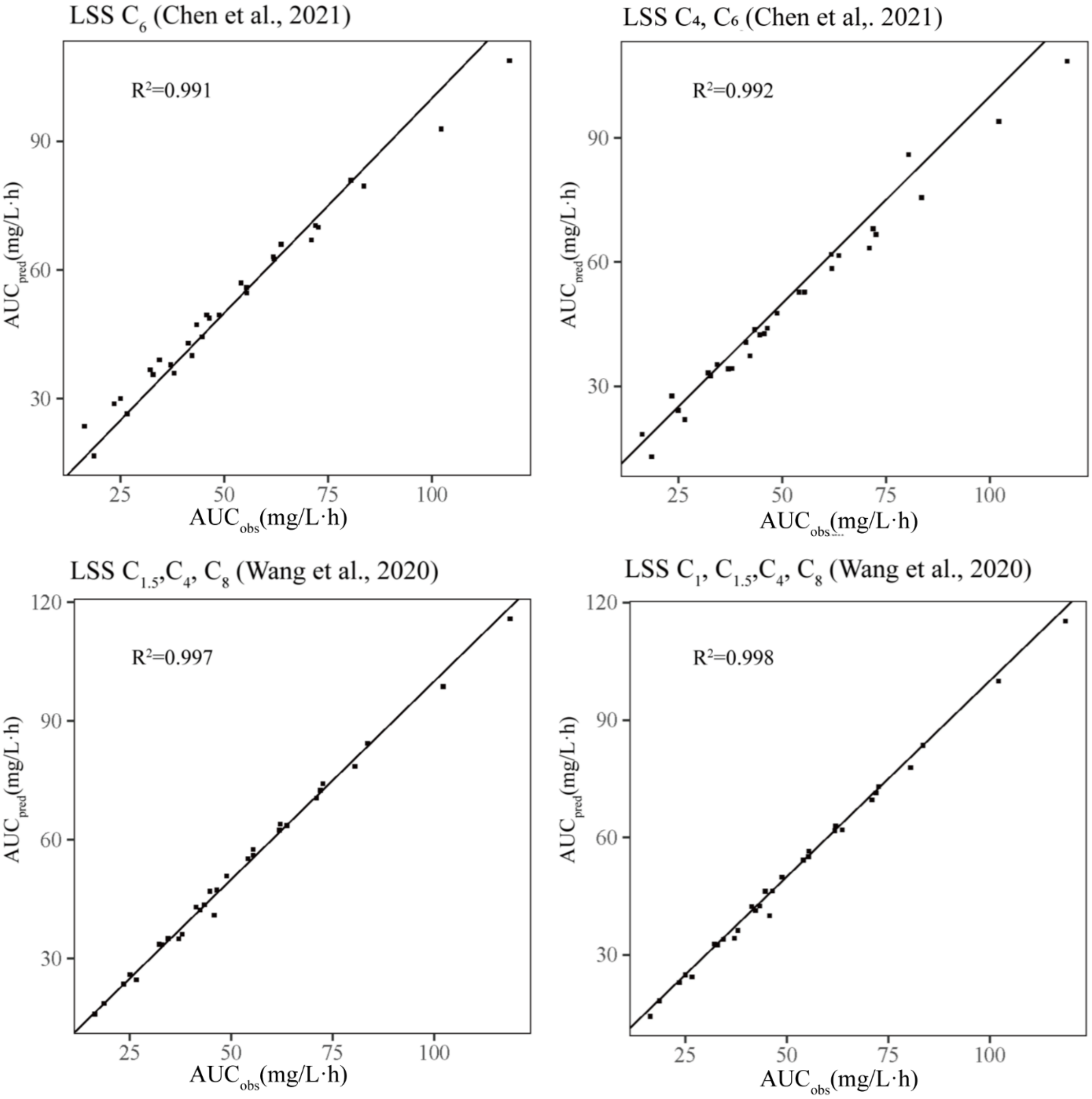

Figure S4. Correlation between AUC_{obs} and $AUC_{pred.}$ AUCpred were estimated from LSS C_6 (Chen et al., 2021), LSS C_4, C_6 (Chen et al., 2021), LSS $C_{1.5}$, C_4, C_6, C_8 (Wang et al., 2020), and LSS C_1, $C_{1.5}$, C_4, C_8 (Wang et al., 2020).

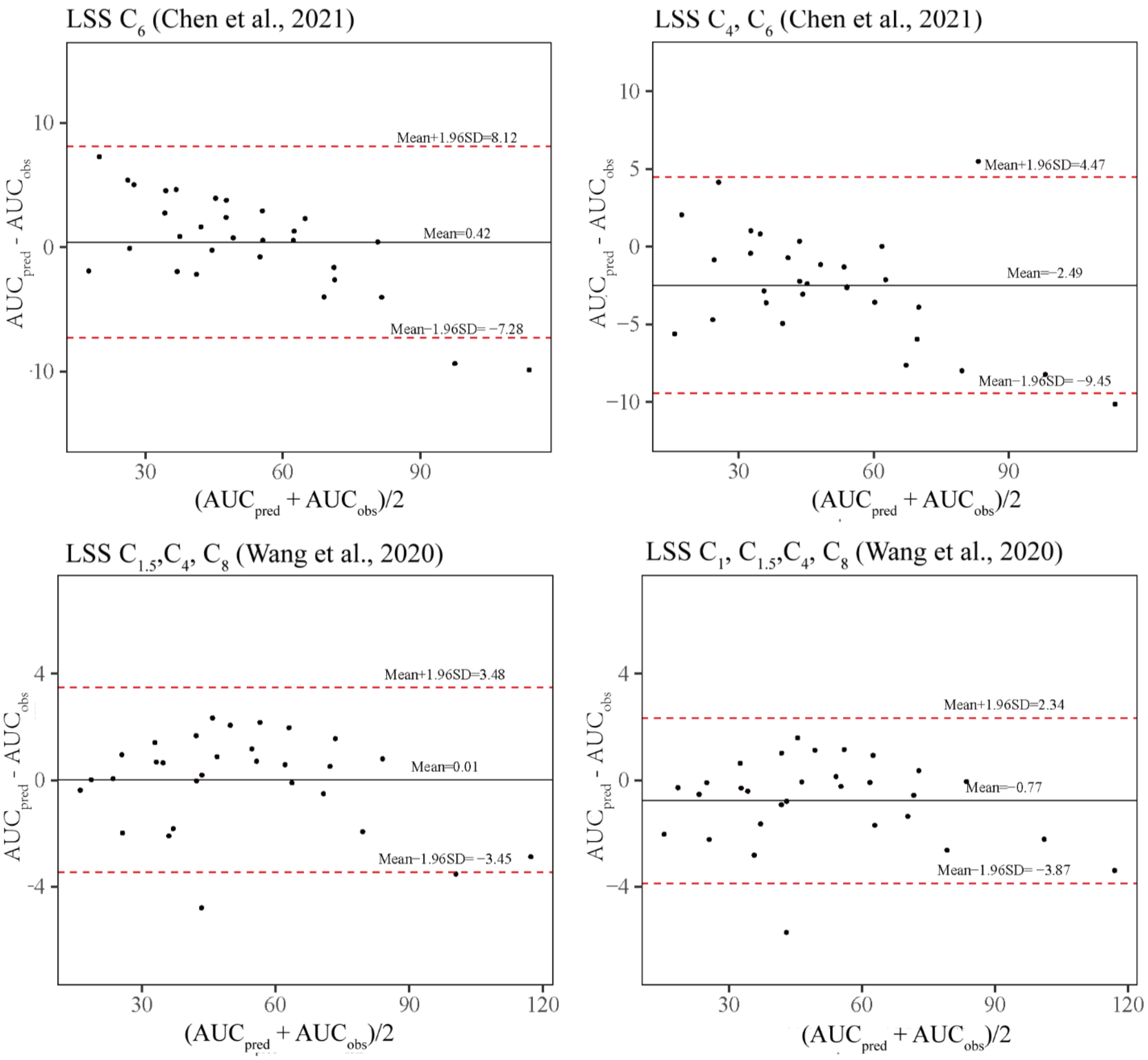

Figure S5. Bland-Altman plots of the relative difference between AUC_{pred} and AUC_{obs} versus the average of AUC_{pred} and AUC_{obs}. AUC_{pred} were estimated from LSS C_6 (Chen et al., 2021), LSS C_4, C_6 (Chen et al., 2021), LSS $C_{1.5}$, C_4, C_6, C_8 (Wang et al., 2020), and LSS C_1, $C_{1.5}$, C_4, C_8 (Wang et al., 2020).

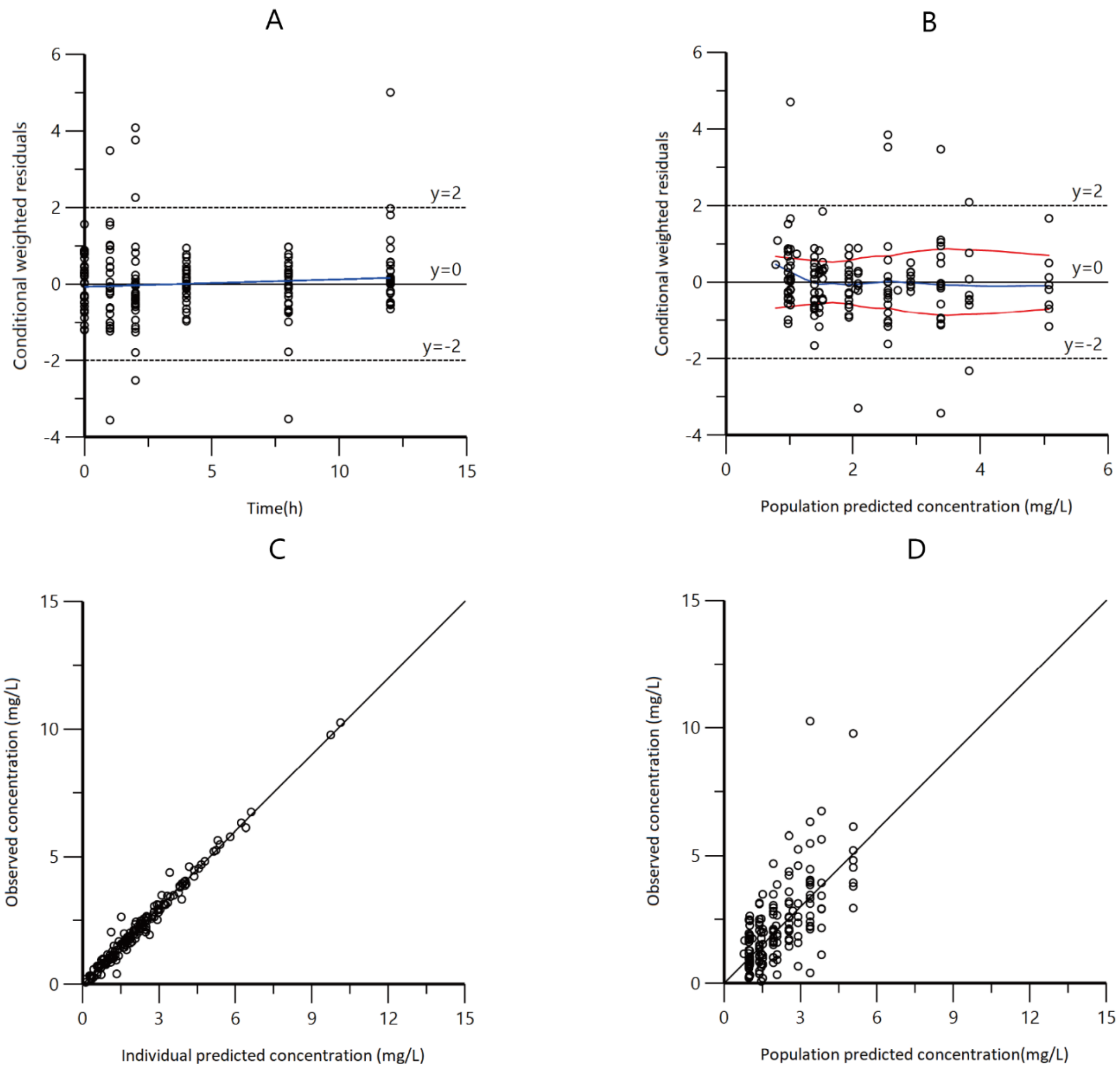

Figure S6. Goodness-of-fit plots for the final population pharmacokinetic model. (A) Conditional weighted residuals versus time (CWRES vs. IVAR); (B) Conditional weighted residuals versus population predicted concentrations (CWRES vs. PRED); (C) Observed versus individual predicted concentrations (DV vs. IPRED); (D) Observed versus population predicted concentrations (DV vs. PRED).

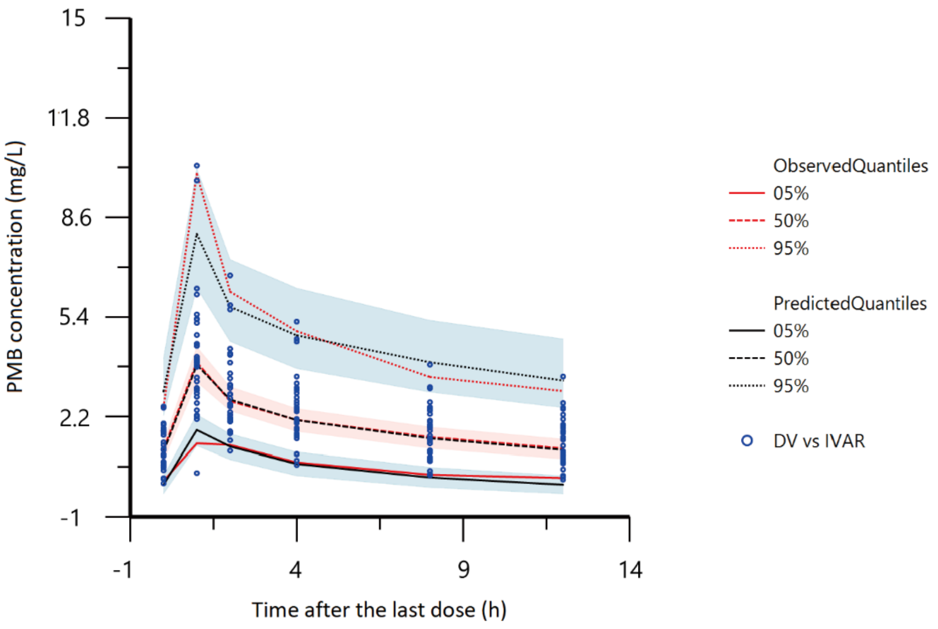

Figure S7. Prediction corrected-visual predictive check of the final model. Red lines represent the 5th, 50th, and 95th percentiles of the observed concentration; the shaded areas represent the 90 % confidence intervals of the 5th, 50th, and 95th percentiles of simulated concentrations, respectively; the dots represent the observed data; DV, PMB observed concentration; IVAR, time after the last dose.

1.2 Supplementary Tables

Table S1. PMB LSSs developed by multiple regression analysis from our center (n= 20)

No.	Time point	Equation	r^2
1	C_0	$34.084\times C_0+10.167$	0.874
2	C_1	$12.062\times C_1+0.745$	0.767
3	C_2	$18.740\times C_2-2.854$	0.943
4	C_4	$20.623\times C_4+2.889$	0.935
5	C_8	$25.549\times C_8+9.340$	0.922
6	C_{12}	$23.949\times C_{12}+17.911$	0.753
7	C_0, C_1	$23.006\times C_0+6.037\times C_1-1.853$	0.974
8	C_0, C_2	$13.469\times C_0+12.633\times C_2-1.440$	0.980
9	C_0, C_4	$10.919\times C_0+14.758\times C_4+3.463$	0.949
10	C_0, C_8	$10.011\times C_0+18.674\times C_8+8.538$	0.931
11	C_0, C_{12}	$25.592\times C_0+7.633\times C_{12}+9.740$	0.896
12	C_1, C_2	$-1.840\times C_1+21.118\times C_2-1.990$	0.946
13	C_1, C_4	$3.356\times C_1+16.334\times C_4-1.142$	0.954
14	C_1, C_8	$4.531\times C_1+18.758\times C_8+1.446$	0.965
15	C_1, C_{12}	$7.422\times C_1+14.235\times C_{12}+0.247$	0.920
16	C_2, C_4	$5.635\times C_2+16.137\times C_4-2.060$	0.973
17	C_2, C_8	$8.661\times C_2+14.564\times C_8+1.740$	0.993
18	C_2, C_{12}	$13.614\times C_2+8.942\times C_{12}-0.910$	0.986
19	C_4, C_8	$11.538\times C_4+12.818\times C_8+3.134$	0.986
20	C_4, C_{12}	$15.604\times C_4+8.476\times C_{12}+2.801$	0.974
21	C_8, C_{12}	$24.469\times C_8+1.253\times C_{12}+9.362$	0.922
22	C_0, C_1, C_2	$18.976\times C_0+3.984\times C_1+4.471\times C_2-1.545$	0.979
23	C_0, C_1, C_4	$15.945\times C_0+4.576\times C_1+6.226\times C_4-1.772$	0.982
24	C_0, C_1, C_8	$14.260\times C_0+5.027\times C_1+8.223\times C_8-0.558$	0.982
25	C_0, C_1, C_{12}	$18.154\times C_0+5.711\times C_1+4.900\times C_{12}-1.477$	0.983
26	C_0, C_2, C_4	$17.147\times C_0+11.868\times C_2-2.398\times C_4+1.222$	0.966
27	C_0, C_2, C_8	$1.198\times C_0+8.922\times C_2+13.439\times C_8+1.452$	0.993
28	C_0, C_2, C_{12}	$7.121\times C_0+10.494\times C_2+7.783\times C_{12}+0.859$	0.989
29	C_0, C_4, C_8	$-6.836\times C_0+13.118\times C_4+15.769\times C_8+2.832$	0.989
30	C_0, C_4, C_{12}	$1.460\times C_0+15.039\times C_4+8.105\times C_{12}+2.882$	0.974
31	C_0, C_8, C_{12}	$10.001\times C_0+17.623\times C_8+1.226\times C_{12}+8.561$	0.931
32	C_1, C_2, C_4	$5.805\times C_1-10.295\times C_2+25.295\times C_4-1.837$	0.960
33	C_1, C_2, C_8	$0.600\times C_1+8.356\times C_2+14.078\times C_8+0.997$	0.993
34	C_1, C_2, C_{12}	$0.776\times C_1+11.400\times C_2+10.303\times C_{12}+0.01$	0.983
35	C_1, C_4, C_8	$2.479\times C_1+9.081\times C_4+11.814\times C_8+0.137$	0.996
36	C_1, C_4, C_{12}	$3.091\times C_1+11.838\times C_4+8.165\times C_{12}-0.908$	0.990
37	C_1, C_8, C_{12}	$4.691\times C_1+15.801\times C_8+15.801\times C_{12}+1.225$	0.968
38	C_2, C_4, C_8	$7.198\times C_2+2.722\times C_4+13.439\times C_8+1.589$	0.994
39	C_2, C_4, C_{12}	$8.611\times C_2+5.015\times C_4+9.436\times C_{12}+0.928$	0.985
40	C_2, C_8, C_{12}	$9.472\times C_2+9.806\times C_8+4.359\times C_{12}+1.146$	0.998
41	C_4, C_8, C_{12}	$11.811\times C_4+9.817\times C_8+3.132\times C_{12}+3.044$	0.988
42	C_0, C_1, C_2, C_4	$15.333\times C_0+5.002\times C_1-1.997\times C_2+8.318\times C_4-1.882$	0.982

Table S1. (Continued)

No.	Time point	Equation	r^2
43	C_0, C_1, C_2, C_8	$4.048\times C_0+1.306\times C_1+7.154\times C_2+11.761\times C_8+0.493$	0.994
44	C_0, C_1, C_2, C_{12}	$10.511\times C_0+2.525\times C_1+6.700\times C_2+6.520\times C_{12}-0.890$	0.994
45	C_0, C_1, C_4, C_8	$0.952\times C_0+2.586\times C_1+8.756\times C_4+11.360\times C_8+0.5$	0.996
46	C_0, C_1, C_4, C_{12}	$7.936\times C_0+3.767\times C_1+7.946\times C_4+6.08\times C_{12}-1.282$	0.995
47	C_0, C_1, C_8, C_{12}	$14.379\times C_{0+}5.199\times C_1+5.02\times C_8+3.320\times C_{12}-0.808$	0.985
48	C_0, C_2, C_4, C_8	$-1.179\times C_0+6.798\times C_2+3.485\times C_4+13.914\times C_8+1.623$	0.994
49	C_0, C_2, C_4, C_{12}	$7.684\times C_0+11.091\times C_2-1.006\times C_4+7.760\times C_{12}+0.813$	0.989
50	C_0, C_2, C_8, C_{12}	$0.688\times C_0+9.402\times C_2+9.443\times C_8+4.334\times C_{12}+1.151$	0.998
51	C_0, C_4, C_8, C_{12}	$-7.422\times C_0+13.553\times C_4+12.736\times C_8+3.428\times C_{12}+2.707$	0.992
52	C_1, C_2, C_4, C_8	$2.151\times C_1+1.313\times C_2+7.799\times C_4+12.060\times C_8+0.252$	0.996
53	C_1, C_2, C_4, C_{12}	$3.094\times C_1-0.14\times C_2+11.851\times C_4+8.163\times C_{12}-0.909$	0.990
54	C_1, C_2, C_8, C_{12}	$0.637\times C_1+8.749\times C_2+9.749\times C_8+4.380\times C_{12}+0.669$	0.998
55	C_1, C_4, C_8, C_{12}	$2.630\times C_1+9.262\times C_4+8.118\times C_8+3.791\times C_{12}-0.154$	0.999
56	C_2, C_4, C_8, C_{12}	$8.194\times C_2+1.871\times C_4+9.463\times C_8+4.238\times C_{12}+1.253$	0.998

Table S2. Predictive performance of selected PMB LSSs developed by MLR analysis from our center

No.	Time point	Equation	MPE %	RMSE %	R	F_{15} %
1	C_0	$34.084×C_0+10.167$	3.08	19.20	0.876	50.00
2	C_1	$12.062×C_1+0.745$	8.12	35.49	0.823	30.00
3	C_2	$18.740×C_2-2.854$	5.09	16.82	0.968	50.00
*4	C_4	$20.623×C_4+2.889$	-2.31	10.60	0.994	90.00
5	C_8	$25.549×C_8+9.340$	0.38	11.96	0.966	80.00
*6	C_0, C_1	$23.006×C_0+6.037×C_1-1.853$	1.53	6.21	0.992	100.00
7	C_0, C_2	$13.469×C_0+12.633×C_2-1.440$	2.96	13.68	0.976	70.00
8	C_2, C_8	$8.661×C_2+14.564×C_8+1.740$	0.27	7.95	0.987	100.00
9	C_2, C_{12}	$13.614×C_2+8.942×C_{12}-0.910$	0.92	11.15	0.980	70.00
10	C_4, C_8	$11.538×C_4+12.818×C_8+3.134$	-2.71	9.41	0.992	90.00
11	C_0, C_2, C_8	$1.198×C_0+8.922×C_2+13.439×C_8+1.452$	0.46	8.40	0.986	80.00
*12	C_1, C_2, C_8	$0.600×C_1+8.356×C_2+14.078×C_8+0.997$	0.44	6.90	0.992	100.00
13	C_1, C_4, C_8	$2.479×C_1+9.081×C_4+11.814×C_8+0.137$	-2.02	6.51	0.998	90.00
14	C_2, C_4, C_8	$7.198×C_2+2.722×C_4+13.439×C_8+1.589$	-0.30	8.06	0.990	90.00
15	C_2, C_8, C_{12}	$9.472×C_2+9.806×C_8+4.359×C_{12}+1.146$	-0.19	8.19	0.986	90.00
16	C_0, C_1, C_4, C_8	$0.952×C_0+2.586×C_1+8.756×C_4+11.360×C_8+0.5$	-0.81	5.73	0.998	90.00
17	C_0, C_2, C_8, C_{12}	$0.688×C_0+9.402×C_2+9.443×C_8+4.334×C_{12}+1.151$	-0.18	8.30	0.985	90.00
*18	C_1, C_2, C_8, C_{12}	$0.637×C_1+8.749×C_2+9.749×C_8+4.380×C_{12}+0.669$	-0.18	6.87	0.991	100.00
19	C_1, C_4, C_8, C_{12}	$2.630×C_1+9.262×C_4+8.118×C_8+3.791×C_{12}-0.154$	-2.61	6.50	0.998	90.00
20	C_2, C_4, C_8, C_{12}	$8.194×C_2+1.871×C_4+9.463×C_8+4.238×C_{12}+1.253$	-0.68	8.03	0.988	90.00

MPE, mean prediction error; RMSE, root mean squared prediction error; R, the Pearson correlation coefficient between AUC_{pred} and $AUC_{obs;}$ F_{15}, the percentage of prediction error falling within the ± 15 %.
*Equation with the best predictability within the same numbers of concentration-time points.

Table S3. A summary of the published PMB LSS studies.

	Country (single/multiple sites)	Diagnosis, number of patients	Age, year	Weight, kg	Daily dose, mg	Infusion duration range, h	Dose frequency	Bioassay	sampling	LSS validation
(Wang et al., 2020)	China(single)	Multidrug-resistant Gram-negative bacterial infections, 37	46 (18-94) [a]	70 (45-98)	100-200	1	q12h	LC-MS/MS, B1+B2, LLQQ:0.2 mg/L, % CV: 13.9 %	C_0, $C_{0.5}$, C_1, $C_{1.5}$, C_2, C_4, C_6 and C_8	Yes, Jackknife method (internal validation)
(Chen et al., 2021)	China(single)	severe pneumonia, 24	63 ±19 [b]	66.1±15.3	100	1	q12h	UPLC-MS/MS, B1+B2+B3, LLQQ: 0.469 mg/L, % CV: 6 %	C_0, $C_{1.5}$, C_2, C_4, C_6, C_8 and C_{12}	Yes, validation in 10 different patients (internal validation)

Abbreviations: q12h, every 12 h; LC-MS/MS, liquid chromatography-tandem mass spectrometry; UPLC-MS/MS, ultra-performance liquid chromatography-tandem mass spectrometry; B1, polymyxin B1; B2, polymyxin B2; LLOQ, lower limit of quantification; % CV, percentage coefficient of variation.

[a] Data represent the median (range).

[b] Data represent the mean ± standard deviation (range).

Table S4. The predictive performance of MLR-based PMB LSSs available in the literature for estimation of PMB AUC_{pred} in patients with MDR Gram-negative bacteria infection treated with PMB.

No.	Time point	Equation	Reference	MPE %	RMSE %	R	F_{15} %
1	C_0	$21.323+28.189\times C_0$	[21][a]	15.53	28.28	0.917	43.33
2	C_2	$-2.099+8.763\times C_2$	[22][b]	-2.04	22.35	0.949	53.33
3	C_4	$0.533+9.876\times C_4$	[22]	-6.64	12.54	0.976	76.67
4	C_4	$-5.601+21.479\times C_4$	[21]	-14.77	19.76	0.976	56.67
*5	C_6	$8.147+21.961\times C_6$	[21]	3.71	11.57	0.993	90.00
6	C_8	$16.168+25.222\times C_8$	[21]	15.98	22.09	0.962	46.67
7	C_{12}	$17.198+30.214\times C12$	[21]	17.98	31.86	0.875	43.33
8	C_0, C_1	$6.811+20.569\times C_0+5.030\times C_1$	[21]	6.18	15.36	0.986	73.33
9	C_0, $C_{1.5}$	$9.349+20.195\times C_0+5.344\times C_{1.5}$	[21]	6.04	14.36	0.980	73.33
10	C_0, C_2	$-0.673+6.048\times C_0+6.203\times C_2$	[22]	0.78	15.19	0.976	76.67
11	C_0, C_2	$3.119+14.215\times C_0+10.228\times C_2$	[21]	-2.52	11.22	0.978	83.33
12	C_0, C_4	$1.608+4.574\times C_0+7.602\times C_4$	[22]	-1.55	11.36	0.979	90.00
13	C_0, C_4	$-0.717+7.805\times C_0+16.225\times C_4$	[21]	-9.39	14.87	0.979	73.33
14	C_0, C_6	$9.303+5.384\times C_0+18.220\times C_6$	[21]	4.42	12.68	0.988	83.33
15	C_2, C_8	$-0.274+4.671\times C_2+7.181\times C_8$	[22]	0.14	10.78	0.991	86.67
*16	C_4, C_6	$2.030+8.532\times C_4+13.465\times C_6$	[21]	-4.34	9.48	0.991	90.00
17	C_4, C_8	$0.196+13.903\times C_4+9.725\times C_8$	[21]	-5.93	10.24	0.993	86.67
18	C_4, C_{12}	$0.546+14.120\times C_4+11.235\times C_{12}$	[21]	-5.33	11.42	0.987	83.33
19	C_1, C_4, C_8	$0.523+0.882\times C_1+4.697\times C_4+6.099\times C_8$	[22]	-1.99	4.68	0.998	96.67
*20	$C_{1.5}$, C_4, C_8	$0.599+1.964\times C_{1.5}+3.169\times C_4+6.633\times C_8$	[22]	0.13	3.63	0.997	100.0
21	C_0, C_4, C_6	$3.467+4.853\times C_0+7.980\times C_4+10.635\times C_6$	[21]	-3.21	9.84	0.988	90.00
22	C_0, C_6, C_8	$7.641+4.158\times C_0+20.045\times C_6-0.526\times C_8$	[21]	3.01	11.32	0.990	83.33
23	C_4, C_6, C_{12}	$2.559+9.518\times C_4+7.739\times C_6+6.894\times C_{12}$	[21]	-3.00	9.16	0.991	93.33
*24	C_1, $C_{1.5}$, C_4, C_8	$0.260+0.460\times C_1+1.137\times C_{1.5}+3.644\times C_4+6.480\times C_8$	[22]	-1.84	4.26	0.998	100.0
25	C_0, C_4, C_6, C_8	$2.879+3.489\times C_0+7.637\times C_4+10.691\times C_6+1.914\times C_8$	[21]	-3.25	9.27	0.991	90.00
26	C_0, C_4, C_6, C_{12}	$3.439+3.541\times C_0+8.801\times C_4+7.503\times C_6+4.696\times C_{12}$	[21]	-2.59	9.30	0.990	90.00

MPE, mean prediction error; RMSE, root mean squared prediction error; R, the Pearson correlation coefficient between AUC_{pred} and $AUC_{obs;}$ F_{15}, the percentage of prediction error falling within the ±15 %.

*Model with the best predictability within the same numbers of concentration-time points.

[a] Prediction of $AUC_{ss,24hr}$.

[b] Prediction of $AUC_{ss,12hr}$.

Table S5. Population PK parameters estimates in the final model and bootstrap

Parameters	Final model results					Bootstrap			
	Estimate	SE	% CV	95 % CI	Shrinkage (%)	Estimate	SE	% CV	95 % CI
tvV (L)	15.6	3.6	23.10	11.89-19.23	NA	15.78	2.54	16.09	9.64-18.02
tvV_2 (L)	11.8	2.82	23.89	8.10-15.53	NA	11.26	3.92	34.77	9.01-22.59
tvCL (L/h)	2.29	0.22	9.66	1.85-2.72	NA	2.27	0.21	9.06	1.89-2.54
$tvCL_2$ (L/h)	6.81	1.59	23.39	3.67-9.95	NA	6.64	1.37	20.63	5.57-7.59
Inter-individual variability									
ω^2V	0.14	0.11	29.73	NA	5.68	0.08	0.05	17.86	NA
ω^2V_2	0.11	0.13	39.39	NA	3.30	0.01	0.05	47.01	NA
ω^2CL	0.07	0.06	22.22	NA	39.91	0.03	0.04	22.22	NA
ω^2CL_2	0.29	0.05	17.24	NA	41.12	0.31	0.07	22.58	NA
Residual variability (σ)									
Proportional error	0.28	0.05	16.59	0.19-0.37	26.1	0.28	0.04	14.43	0.20-0.33

SE, standard error; CV, percent confidence of variation; Cl, confidence interval; tvV, typical value of volume of central compartment distribution (V); tvV_2, typical value of volume of peripheral compartment distribution (V_2); tvCl, typical value of central compartment clearance (Cl); $tvCl_2$, typical value of inter-compartmental clearance (Cl_2); ω, interindividual variation; NA, not applicable.

Table S6. Individual pharmacokinetic parameter estimates

ID	V (L)	V_2 (L)	Cl (L/h)	Cl_2 (L/h)
1	16.31	14.15	2.37	7.01
2	25.53	11.21	2.14	6.80
3	7.06	10.48	1.22	6.47
4	8.38	15.16	1.05	6.99
5	9.75	18.32	1.26	7.01
6	12.85	14.20	3.57	6.83
7	20.74	11.32	3.07	6.87
8	17.13	10.41	2.73	6.84
9	21.78	13.05	1.54	6.86
10	11.32	16.44	8.54	7.01
11	18.28	12.47	1.46	6.89
12	2.68	6.72	0.98	6.25
13	6.74	10.58	3.64	6.56
14	25.23	10.57	2.78	6.78
15	25.77	12.58	4.51	6.85
16	45.15	10.25	5.63	6.78
17	12.12	3.44	1.82	6.99
18	16.73	10.22	1.50	6.87
19	18.82	6.09	1.48	6.77
20	26.39	12.72	2.97	6.88
21	17.97	15.18	1.96	6.94
22	26.80	14.39	3.82	6.88
23	12.51	10.35	2.30	6.88
24	27.14	22.27	1.71	7.00
25	20.75	9.31	4.00	6.84
26	10.38	12.74	2.64	6.65
27	26.58	11.27	1.60	6.84
28	16.57	12.62	2.00	6.80
29	13.72	11.35	1.75	6.78
30	5.68	9.67	2.63	6.67

V, volume of central compartment distribution; V_2, volume of peripheral compartment distribution; Cl, central compartment clearance; Cl_2, inter-compartmental clearance.

Table S7. The predictive performance of the validated LSSs from the published literature following the simulation under infusion time of 0.5 h, 1.5 h, 2 h, and 2.5 h.

No.	Time point	Reference	MPE %					RMSE %				
			0.5 h	1 h	1.5 h	2 h	2.5 h	0.5 h	1 h	1.5 h	2 h	2.5 h
3	C_4	[22]	-5.92	-6.64	-0.79	2.36	6.19	19.44	12.54	19.59	20.36	21.94
5	C_6	[21]	4.77	3.71	8.53	10.56	12.73	20.28	11.57	22.32	23.61	25.12
8*	C_0, C_1	[21]	3.89	6.18	0.18	-4.53	-6.86	17.92	15.36	16.42	15.96	16.53
9	C_0, $C_{1.5}$	[21]	5.04	6.04	15.52	8.33	4.51	19.05	14.36	26.31	20.65	18.47
10	C_0, C_2	[22]	-0.76	0.78	11.52	22.84	9.79	17.97	15.19	23.83	35.48	23.77
11	C_0, C_2	[21]	1.76	-2.52	12.26	21.79	11.23	17.69	11.22	23.59	33.10	23.47
12	C_0, C_4	[22]	-0.79	-1.55	3.97	6.82	10.20	18.46	11.36	19.64	20.84	22.67
13*	C_0, C_4	[21]	-8.63	-9.39	-3.72	-0.77	2.75	20.20	14.87	19.20	19.18	19.74
14	C_0, C_6	[22]	5.43	4.42	9.02	10.96	13.01	20.41	12.68	22.38	23.62	25.05
15	C_2, C_8	[22]	2.31	0.14	12.69	21.82	12.62	18.83	10.78	23.93	32.34	24.13
16	C_4, C_6	[21]	-3.38	-4.34	1.14	3.74	6.73	18.92	9.48	19.48	20.27	21.53
17	C_4, C_8	[21]	-3.03	-5.93	1.90	4.81	8.22	19.32	10.24	19.85	20.72	22.17
18*	C_4, C_{12}	[21]	-5.87	-5.33	-1.16	1.63	4.93	19.06	11.42	18.59	18.86	19.67
19*	C_1, C_4, C_8	[22]	0.39	-1.99	2.54	2.92	4.48	18.02	4.68	18.45	18.79	19.46
20	$C_{1.5}$, C_4, C_8	[22]	2.56	0.13	12.38	8.37	7.06	18.56	3.63	23.35	20.67	20.04
21	C_0, C_4, C_6	[21]	-2.29	-3.21	2.03	4.51	7.34	18.47	9.84	19.20	20.04	21.30
22	C_0, C_6, C_8	[21]	3.94	3.01	7.67	9.68	11.82	19.70	11.32	21.52	22.72	24.13
23	C_4, C_6, C_{12}	[21]	-2.92	-3.00	1.51	4.08	7.03	18.39	9.16	18.85	19.57	20.75
24*	C_1, $C_{1.5}$, C_4, C_8	[22]	0.50	-1.84	6.83	4.42	4.15	18.06	4.26	19.88	18.88	18.92
25	C_0, C_4, C_6, C_8	[21]	-1.90	-3.25	2.48	4.98	7.83	18.67	9.27	19.47	20.34	21.64
26	C_0, C_4, C_6, C_{12}	[21]	-2.26	-2.59	2.06	4.54	7.39	18.26	9.30	18.88	19.66	20.88

MPE, mean prediction error; RMSE, root mean squared prediction error. *Equation with the best predictability within the same numbers of concentration-time points.

Table S7. (Continued)

No.	Time point	Reference	R					F_{15} %				
			0.5 h	1 h	1.5 h	2 h	2.5 h	0.5 h	1 h	1.5 h	2 h	2.5 h
3	C_4	[22]	0.937	0.976	0.936	0.936	0.935	76.67	76.67	83.33	73.33	83.33
5	C_6	[21]	0.939	0.993	0.939	0.939	0.939	80.00	90.00	73.33	76.67	70.00
8*	C_0, C_1	[21]	0.950	0.986	0.952	0.950	0.947	76.67	73.33	76.67	73.33	63.33
9	C_0, $C_{1.5}$	[21]	0.946	0.980	0.953	0.952	0.950	76.67	73.33	63.33	63.33	76.67
10	C_0, C_2	[22]	0.948	0.976	0.944	0.930	0.941	83.33	76.67	76.67	66.67	76.67
11	C_0, C_2	[21]	0.949	0.978	0.948	0.940	0.948	83.33	83.33	73.33	80.00	73.33
12	C_0, C_4	[22]	0.943	0.979	0.944	0.944	0.944	80.00	90.00	80.00	83.33	80.00
13*	C_0, C_4	[21]	0.942	0.979	0.943	0.943	0.943	73.33	73.33	80.00	73.33	83.33
14	C_0, C_6	[22]	0.939	0.988	0.940	0.940	0.941	80.00	83.33	73.33	56.67	66.67
15	C_2, C_8	[22]	0.947	0.991	0.946	0.940	0.946	83.33	86.67	80.00	80.00	76.67
16	C_4, C_6	[21]	0.940	0.991	0.940	0.940	0.940	80.00	90.00	80.00	80.00	80.00
17	C_4, C_8	[21]	0.942	0.993	0.942	0.942	0.942	76.67	86.67	83.33	83.33	80.00
18*	C_4, C_{12}	[21]	0.945	0.987	0.946	0.947	0.948	73.33	83.33	76.67	83.33	83.33
19*	C_1, C_4, C_8	[22]	0.946	0.998	0.947	0.946	0.946	83.33	96.67	83.33	83.33	80.00
20	$C_{1.5}$, C_4, C_8	[22]	0.946	0.997	0.948	0.948	0.948	83.33	100.00	80.00	80.00	83.33
21	C_0, C_4, C_6	[21]	0.941	0.988	0.942	0.942	0.942	73.33	90.00	80.00	73.33	80.00
22	C_0, C_6, C_8	[21]	0.939	0.990	0.940	0.940	0.940	76.67	83.33	76.67	80.00	73.33
23	C_4, C_6, C_{12}	[21]	0.943	0.991	0.944	0.945	0.945	73.33	93.33	80.00	86.67	80.00
24*	C_1, $C_{1.5}$, C_4, C_8	[22]	0.946	0.998	0.948	0.948	0.947	83.33	100.00	86.67	80.00	86.67
25	C_0, C_4, C_6, C_8	[21]	0.941	0.991	0.942	0.942	0.942	73.33	90.00	80.00	80.00	80.00
26	C_0, C_4, C_6, C_{12}	[21]	0.942	0.990	0.944	0.944	0.945	70.00	90.00	80.00	80.00	80.00

R, the Pearson correlation coefficient between AUC_{pred} and AUC_{obs}; F_{15}, the percentage of prediction error falling within the ± 15 %. *Equation with the best predictability within the same numbers of concentration-time points.

Table S8. The predictive performance of the LSSs (C_0, C_1) that were established by our center following the sampling time error of 0, ± 5, ± 10, ± 15, ± 20, ± 25, and ± 30min.

Sampling error time (min)	MPE %	RMSE %	R	F_{15} %
-30	-15.58	18.09	0.985	50.00
-25	-12.63	15.53	0.986	76.67
-20	-9.51	13.13	0.987	80.00
-15	-6.87	11.52	0.988	86.67
-10	-4.35	10.48	0.988	90.00
-5	-1.92	10.07	0.988	90.00
0	1.46	8.57	0.988	90.00
5	-1.56	9.31	0.989	93.33
10	-3.68	9.44	0.988	90.00
15	-5.39	10.28	0.986	90.00
20	-6.93	11.44	0.984	86.67
25	-8.47	12.87	0.982	76.67
30	-9.70	14.13	0.980	76.67

MPE, mean prediction error; RMSE, root mean squared prediction error; R, the Pearson correlation coefficient between AUC_{pred} and AUC_{obs}; F_{15}, the percentage of prediction error falling within the ± 15 %.
